# Status of knowledge, attitude and practice of clinical nurses towards the intrahospital transport of critically ill patients: A cross‐sectional study

**DOI:** 10.1002/nop2.2172

**Published:** 2024-06-05

**Authors:** Jie Dong, Yayan Yi, Xiaocha Zhu, Huafang Zhang

**Affiliations:** ^1^ Department of Nursing, the Fourth Affiliated Hospital Zhejiang University School of Medicine Yiwu China

**Keywords:** attitude, behaviour, critically ill patients, intrahospital transport, knowledge, nurse

## Abstract

**Aims:**

To explore the knowledge, attitudes and practice status of the intrahospital transport (IHT) of critically ill patients among clinical nurses and their influencing factors.

**Design:**

Cross‐sectional study.

**Methods:**

A questionnaire determined the nurses' knowledge, attitudes and practice scores. The questionnaire was used for data collection in a tertiary hospital from 10 January to 17 January 2023. Multivariate regression analysis was also used to evaluate the related factors of IHT of critically ill patients in different dimensions.

**Results:**

Out of 670 distributed questionnaires, 612 nurses returned the completed questionnaire. The scores of KAP were (9.72 ± 1.61), (42.91 ± 4.58) and (82.84 ± 1.61), respectively. Pearson's correlation analysis showed that knowledge, attitude and behaviour scores were positively correlated. Variables that were associated with the scores of transfer knowledge were the scores of transfer practice, different departments and the scores of transfer attitude. The score of practice, number of IHT and received hospital‐level training had statistical significance on the nurses' attitude scores. Furthermore, the score of the attitude and transport knowledge had statistical significance on the nurses' practice.

**Conclusion:**

The findings indicate a clear need for clinical nurses' knowledge of IHT of critically ill patients, especially in the emergency department (ED) and ICU. In addition, nurses need to be more active in transporting critically ill patients. Managers should enhance nurses' confidence in the IHT of critically ill patients and promote clinical nurses to establish a correct and positive attitude.

**Impact:**

The findings of this study benefit nursing managers in understanding the current situation of IHT of critically ill patients. Managers should apply new training methods to nursing education and develop a multi‐level training program that is systematic, comprehensive and demand‐oriented.

**Patient or Public Contribution:**

The participants of this study were nurses and this contribution has been explained in the Data collection section. There was no patient contribution in this study.

## 1 INTRODUCTION

Intrahospital transport (IHT) is the transport of patients between different units in the hospital (Haydar et al., 2019). Critically ill patients are defined as patients whose vital signs are unstable, whose condition is complex, serious and changing rapidly and whose lives may be in danger at any time (Veiga et al., 2019). However, to further clarify the diagnosis or treatment needs of critically ill patients, IHT is inevitable (Haydar et al., 2019). Due to various factors such as members of the transport team, equipment and environment involved in the IHT process, IHT becomes a complex, high‐risk procedure that can directly or indirectly cause adverse events (AEs) (Hu et al., 2021). According to a literature report (Venkategowda et al., 2014), the total incidence of transport‐related AEs is 21.2%–67.9% and the incidence of serious AEs that require interventions such as administration of vasopressors, fluid boluses or even CPR may be as high as 4.2%–9.1%. However, according to a recent systematic review and meta‐analysis (Murata et al., 2022), AEs during IHT have been reported to occur in 6%–70% of cases. Although reported AE occurrence rates in IHT vary widely. This may be related to the difference of nursing quality and the different definitions of AEs. The most common AEs are severe hypotension, decreased consciousness requiring intubation, increased intracranial pressure and respiratory cardiac arrest (An et al., 2022). In addition, AEs can threaten the patient's safety, lead to a deterioration of the patient's clinical condition (Ling et al., 2023; Parmentier‐Decrucq et al., 2013) and increase the length of hospitalisation duration and cost (Ling et al., 2023; Schwebel et al., 2013). In all published guidelines, the role of the medical practitioners, specifically the nurse, in preventing possible complications is pivotal (Critical Care Medicine Branch of Chinese Medical Association., 2010; Warren et al., 2004; Whiteley et al., 2011; Williams et al., 2020). To ensure the timeliness and safety of IHT for these patients, it is urgent to standardise and optimise the IHT process (Lu et al., 2022) and and to investigate the knowledge, attitude and practice (KAP) of nurses in this process (Alamanou & Brokalaki, 2014).

### 1.1 Background

As the direct caregivers of patients, nurses play an essential role in all key links of IHT of critically ill patients (Sharafi et al., 2020). Nurses are actively involved in intrahospital transportation from planning, equipment preparation and manipulation and evaluation of the process. By identifying AEs and modifying risk factors, nurses can improve patients' safety as they provide continuous care and are close to the patient (Sharafi et al., 2020). Nurses' knowledge, positive nursing attitude and standardised nursing behaviour can significantly improve the safe transportation of critically ill patients (Shields et al., 2015; Song et al., 2022). Despite nurses playing a vital role in IHT, the previous studies primarily focus on procedures, equipment and guidelines of IHT. Thus, our current understanding of clinical nurses' knowledge, attitude and behaviour regarding IHT of critically ill patients is limited. Additionally, the development of medical resources in hospitals in different cities is unbalanced and nurses' levels of knowledge, attitude and behaviour are different.

In recent years, most of the studies on the IHT of critically ill patients have been performed in the ICU and the emergency department (ED) and few have been performed in other departments. According to a cross‐sectional study, a total of 480 nurses from 12 adult intensive care units in China participated, 75% of respondents expressed that IHT was a source of stress and increased workload (Song et al., 2022). This stress and workload could negatively affect their performance and threaten the patient's safety. Additionally, Hu et al. (2021) conducted a cross‐sectional study on the current status of IHT of critically ill patients and emergency nurses’ perception of IHT across China. The study found that some nurses in emergency rooms were still unaware of the risks of IHT; 19.3% considered all nurses competent enough to perform IHT of critically ill patients without special training and 7.6% even considered nursing students capable of the task. Moreover, research from O'Leary et al. (2018) has confirmed that it is not uncommon for nurses with inadequate supervision and training to participate in the IHT. Several studies have been conducted on the risks associated with IHT and checklists have been developed based on evidence and expert opinions (Bergman et al., 2020; Brunsveld‐Reinders et al., 2015; Murata et al., 2022). However, the percentage of aEs has not yet been reduced to zero (Hashemian et al., 2023). Therefore, pursuing a study on nurses’ level of knowledge, attitude and behaviour is essential to inform any efforts to promote critically ill patient safety during IHT.

This study investigated the status of clinical nurses’ knowledge, attitude and practice about IHT of critically ill patients and identified influencing factors. The findings from this study will help identify gaps in nurses’ knowledge and competencies, thereby providing insights into developing future IHT education and practice interventions.

## 2 THE STUDY

### 2.1 Aims

This study explored nurses’ knowledge, attitude and practice in IHT of critically ill patients. The objectives were to: examine the status of clinical nurses’ knowledge, attitude and practice about IHT of critically ill patients.

### 2.2 Design

A cross‐sectional design using an anonymous online survey was conducted.

### 2.3 Samples and setting

The statistical population of this study includes all nurses from a tertiary hospital in Zhejiang, China. This tertiary hospital in China is a general hospital with a bed capacity exceeding 1200, providing specialist health services and serving as a medical hub for multiple regions. This hospital receives national and international patients and it has a mandate to treat patients and serve as a teaching hospital. The clinical nurses who volunteered to participate in this study in the hospital were selected using the convenient sampling method. Participants' eligibility criteria were as follows: (1) registered nurse; (2) clinical working time ≥0.5 years; (3) volunteer to participate in this study. Exclusion criteria were: (1) nurses from other hospitals who come to our hospital for further study and student nurses; (2) nurses who do not participate in the IHT of critically ill patients; (3) incomplete information.

The sample size was computed using the statistical formula of Yamane (1967), which states that, for any given population, the required sample size is calculated by:

$$n=\frac{N}{1+N\left(e\right)2}$$

where: *n* = the required sample size; *N* = the known population size; and e = the margin error, which is =0.05. Finally, 612 nurses were included in this study.

### 2.4 Data collection

Data were collected during 10–17 January 2023. Participants were recruited through a three‐step process: (1) An email invitation to the study was first sent to the nursing leaders, comprising a letter to invite all registered nurses to participate and the survey's hyperlink to access the survey. (2) The nursing leaders disseminated the study invitation to the head nurses. (3) The head nurses disseminated the study invitation to the nurses. The online survey was collected using ‘Wen juanxin’ and piloted to ensure user‐friendliness, ease of electronic interface and effective response collection.

### 2.5 Instruments

#### 2.5.1 Demographic and work‐related characteristics form

Information on the general characteristics of the participants (department, gender, age, education level, general nursing experience, professional title, job grade) and work‐related characteristics (whether they had participated in‐hospital transport of critically ill patients, received IHT education or training, method of acquiring knowledge, whether the condition of critically ill patients has deteriorated during IHT and technology‐related or other AEs have occurred during IHT) was collected using a self‐report questionnaire.

#### 2.5.2 Questionnaire in assessing nurses's knowledge, attitude and practice of the IHT

The questionnaire mainly referred to the scale compiled by Brunsveld‐Reinders et al. (2015) and Mukabagire (2019) was developed through a literature review, combined with several guides (Warren et al., 2004; Whiteley et al., 2011), literature (Brunsveld‐Reinders et al., 2015; Song et al., 2022) and several guides and literature (Brunsveld‐Reinders et al., 2015; Song et al., 2022; Warren et al., 2004; Whiteley et al., 2011). To use the questionnaire, the questionnaire's principal designer's permission was obtained, following which the tool underwent a forward and backward translation process. The English translation and the original version were then compared by independent translators. Finally, based on the theory of KAP (Bettinghaus, 1986), a group of eight experienced nurses evaluated the translated versions' relevance and utility. Some questions were removed, others adapted. The formal questionnaire of this study was developed after two rounds of expert letters and pre‐investigation. Notably, a 42‐item questionnaire was created. The questionnaire consists of three dimensions: KAP. Before the formal investigation, 20 nurses were randomly selected for pre‐investigation. The content validity of the final version of the questionnaire (I‐CVI) had a value of 0.781, the Cronbach's a for subscales ranged from 0.751 to 0.823 and the Kaiser‐Meyer‐Olkin (KMO) value was 0.71, with good reliability and validity. The dimension of knowledge includes 13 items, 5 single‐choice and 8 multiple‐choice questions and the total score is 13 points. The higher the score, the higher the nurses' cognitive level of IHT of critical patients. The dimensions of attitude and practice were evaluated by a 5‐point Likert scale. The dimension of attitude includes 11 items, with scores ranging from 11 to 55. The higher the score, the more positive the nurses' transport attitude towards critical patients and the higher their willingness to accept it. The dimension of practice includes 18 items with scores ranging from 18 to 90. The higher the score, the more standardised the behaviour of nurses when transporting critically ill patients. Scoring rate = average score/perfect score *100%, scoring rate >80% is good (positive), scoring rate 60%–80% is medium (neutral) and scoring rate < 60% is poor (negative) (Mukabagire, 2019).

### 2.6 Ethical review

This study was approved by the Zhejiang University School of Medicine the Fourth Affiliated Hospital Research Ethics Committee (K2023043). Participation in the survey occurred voluntarily and completing and submitting the online survey implied the participant's consent. No personally identifiable data was collected. Confidentiality and anonymity about the survey responses were assured for all the participants.

### 2.7 Data analysis

For all statistical analyses, the IBM SPSS Statistics for Windows Version 23.0 was used (IBM Corporation). Mean and standard deviations (SD), proportions and percentages were computed to summarise the participants' demographic characteristics, knowledge, attitude and behaviour scores. Independent sample *t*‐test and one‐way analysis of variance (ANOVA) were used to examine the differences in nurses' knowledge, attitude and behaviour scores among the various categorical demographics.

Pearson product‐moment correlation coefficients were calculated to examine the correlation between knowledge, attitude and behaviour. To explore factors influencing nurses' knowledge, attitude and behaviour scores (dependent variable), the variables found to be statistically significant in the univariate linear regression analyses were used as independent variables in the subsequent multiple linear regression analysis. In all other analyses, the level of statistical significance was set at 0.05.

## 3 RESULTS

### 3.1 Nurse characteristics

Out of 670 questionnaires distributed, 612 nurses returned questionnaires completed, corresponding to a response rate of 91.3%. The demographic characteristics of the participants are presented in Table 1. The purpose for IHT includes 41.3% being transferred from the ward to ICU, 39.4% operated on or intervened, 11.3% transferred from the emergency room to the ward, 11.3% transferred from the emergency room to ICU and 18.6% transferred from ICU to ward. The first three types of AEs related to the deterioration of critically ill patients during IHT were the SPO_2_ decreases by≥5% or <90% (9.7%), consciousness change (4.4%) and changes in respiratory frequency and respiratory pattern (4.4%). Furthermore, the first three types of AEs involving technology related to critically ill patients during IHT were insufficient oxygen supply (4.4%), monitor disconnection (3.6%) and insufficient battery shortage of transhipment instruments and equipment (3.4%).

**TABLE 1:** Demographics characteristics of nurses.

Characteristics	Total, *n* (%)	
Departments	Emergency room	57 (9.3)
	Special department	93 (15.2)
	ICU	110 (18)
	Department of surgery	166 (27.1)
	Department of internal medicine	153 (25.0)
	Maternity department	33 (5.4)
Gender	Men	90 (14.7)
	Female	522 (85.3)
Age	20–29	449 (73.4)
	30 and above	163 (26.6)
Education level	Associate degree	12 (2.0)
	Bachelor of Nursing and above	600 (99.8.)
Years of nursing practice	0.5–1 year	95 (15.5)
	1–2 years	133 (21.7)
	3–5 years	172 (28.1)
	More than 6 year	212 (346)
Job grade	N0	124 (20.3)
	N1	166 (27.1)
	N2	238 (38.9)
	N3 and above	84 (12.9)
Professional title	Staff nurse	134 (21.9)
	Senior nurse	339 (55.4)
	Supervisor nurse and above	139 (22.7)
Received ITH education or training	No	63 (10.3)
	Yes	549 (89.7)
Training methods	Theoretical teaching in departments	475 (77.6)
	Emergency drills in clinical departments	403 (65.8)
	Hospital‐level training	296 (48.4)
Number of IHT	0–5 times	455 (74.3)
	6 times and above	157 (25.7)

*Note*: Special departments: Endoscopy room, Haemodialysis room, Operating room. N0: A newly graduated nurse who has joined the work. N1: Nurses who have worked in clinical practice for 1 year or more, obtained college education or above and obtained the qualification certificate of nurses. It is also necessary to meet the necessary requirements. N2: Nurses who have worked in clinical practice for 3 years or more and have the title of junior nurse or above. It is also necessary to meet the necessary requirements. N3: Nurses who are in charge of nurses and above. It is also necessary to meet the necessary requirements.

### 3.2 The level of knowledge, attitude and practice of nurses on intrahospital transportation

The scores of 612 nurses' KAP about IHT of critically ill patients were as follows: (1) The score range of knowledge dimension was 2–13 (9.72 ± 1.61). The scoring rate was 74.7%, of which 201 (32.8%) were reported to have a high level of knowledge, 354 (57.8%) were classified as having a moderate level and 57 (9.4%) were categorised as having a low level of knowledge. (2)The score range of attitude dimension was 26–55 (40.44 ± 3.67) and the scoring rate was 73.5%, of which 226 (36.9%) had positive attitudes, 374 (61.1%) had neutral attitudes, 12 (2.0%) had negative attitudes. (3) The score range of practice dimension was 18–90 (82.84 ± 1.61) and the scoring rate was 92.04%, among which 496 (80.7%) were classified as high, 114 (19.0%) were classified as medium and 2 (0.3%) were classified as low level of practice. The three items with the highest and lowest scores on nurses' KAP about IHT of critically ill patients are presented in Table 2.

**TABLE 2:** The three items with the highest and lowest scores on KAP regarding the transport of critically ill patients.

Dimension	The item with the highest score	Score ($\bar{X}$ ± s)	The item with the lowest score	Score ($\bar{X}$ ± s)
Knowledge dimension	1. Preparation of stuff before IHT	0.96 ± 0.20	1. Ventilation maintenance in critically ill patients' examination	0.45 ± 0.50
	2. Evaluation content before IHT	0.94 ± 0.24	2. Knowledge of oxygen therapy during IHT	0.32 ± 0.47
	3. Administration of medication during transportation	0.94 ± 0.25	3. Informed person when leaving the department for transport	0.25 ± 0.43
Attitude dimension	1.No drug was lost during IHT	4.55 ± 0.65	1. I have never been confident in transporting patients because I have never had any training on it, even in‐service	3.82 ± 0.96
	2. I can't understand so much about the pre‐transport preparation of the critically ill patient.	4.48 ± 0.85	2. One never finds time to prepare for the transport of patients because everything is done in a rush	3.73 ± 0.96
	3. The report we receive about transfers is very comprehensive and directive to the continuity of care	4.40 ± 0.64	3. I fear transporting critically ill patients because we have had many adverse events during patient transportation	3.26 ± 0.78
Practice dimension	1. Notify the receiving department to take isolation measures before IHT	4.75 ± 0.53	1. Before IHT, the signing of the informed consent form will be carefully confirmed	4.51 ± 0.78
	2. Evaluate drug use before transport	4.71 ± 0.52	2. After IHT, the work was comprehensively evaluated	4.46 ± 0.87
	3. Always pay attention to the patient's symptoms and signs, whether to maintain the best position and pipeline	4.70 ± 0.52	3. Nurses know the transfer route and the area where first aid equipment can be obtained	4.38 ± 0.86

### 3.3 Correlation analysis of knowledge, attitude and practice of IHT of critically ill patients

Pearson correlation analysis showed a positive correlation between knowledge and attitude scores (*r* = 0.204, *p* < 0.01). Notably, the scores of knowledge are positively correlated with practice (*r* = 0.206, *p* < 0.01) and the scores of practice are positively correlated with attitude (*r* = 0.411, *p* < 0.01).

### 3.4 Comparison of KAP scores of nurses with different characteristics on IHT of critically ill patients

Using nurses' general data as independent variables, this study analysed the differences in knowledge, attitude and practices of nurses with different characteristics about IHT of critically ill patients. Important differences in nurses' knowledge scores were observed between, different departments, the number of IHT, received emergency drills in clinical departments, received hospital‐level training and gender (*p* < 0.05). Different departments, job grades, ages, professional titles, years of nursing practice, received IHT education or training, receive emergency drills in clinical departments, receive hospital‐level training and number of IHT had statistical significance on the nurses' attitudes (*p* < 0.05). There are important differences in practice scores between different departments and the number of IHT (*p* < 0.05), as shown in Table 3.

**TABLE 3:** Univariate analysis of influencing nurses' knowledge, attitude and practice about the transport of critically ill patients (*n* = 612).

Variables		*n*	Knowledge dimension			Attitude dimension			Practice dimension		
			Score ($\bar{X}$ ± s)	Statistics	*p*	Score ($\bar{X}$ ± s)	Statistics	*p*	Score ($\bar{X}$ ± s)	Statistics	*p*
Departments	ICU and Emergency room	167	9.40 ± 1.62	*t* = −3.07	0.002	43.59 ± 4.80	*t* = 2.24	0.025	84.04 ± 8.57	*t* = 2.06	0.040
	Other departments	445	9.84 ± 1.59			42.66 ± 4.48			82.38 ± 8.95		
Gender	Men	90	9.40 ± 1.71	*t* = −2.04	0.041	43.11 ± 5.22	*t* = 0.43	0.664	84.04 ± 8.45	*t* = −1.40	0.163
	Female	522	9.77 ± 1.58			42.88 ± 4.47			82.63 ± 8.94		
Age	20–29	449	9.73 ± 1.66	*t* = 0.35	0.725	42.62 ± 4.76	*t* = −2.65	0.008	82.82 ± 9.13	*t* = −0.08	0.934
	30 and above	163	9.68 ± 1.45			43.73 ± 3.94			82.89 ± 8.15		
Education level	Associate degree	12	9.67 ± 1.78	*t* = −0.11	0.909	43.75 ± 4.11	*t* = 0.64	0.526	84.17 ± 8.74	*t* = 0.52	0.909
	Bachelor of Nursing and above	600	9.72 ± 1.60			42.90 ± 4.60			82.81 ± 8.88		
Years of nursing practice	0.5–1 year	95	9.49 ± 1.54	*F* = 1.13	0.336	41.04 ± 5.02^c^	*F* = 8.64	<0.001	83.06 ± 8.86	*F* = 0.23	0.875
	1–2 years	133	9.63 ± 1.90			42.78 ± 5.01^b^			82.35 ± 9.12		
	3–5 years	172	9.83 ± 1.60			42.88 ± 4.40^b^			82.76 ± 9.85		
	More than 6 year	212	9.79 ± 1.43			43.86 ± 3.98^a^			83.12 ± 7.88		
Job grade	N0	124	9.44 ± 1.64^b^	*F* = 1.91	0.127	41.34 ± 4.97^c^	*F* = 8.24	<0.001	82.90 ± 8.79	*F* = 0.47	0.701
	N1	166	9.83 ± 1.76^a^			42.67 ± 4.93^b^			82.41 ± 10.14		
	N2	238	9.28 ± 1.53^a^			43.55 ± 4.21^ab^			83.31 ± 8.13		
	N3 and above	84	9.64 ± 1.39^ab^			43.94 ± 3.66^a^			82.26 ± 8.41		
Professional title	Staff nurse	134	9.50 ± 1.66^b^	*F* = 1.98	0.138	41.46 ± 4.96^b^	*F* = 10.20	<0.001	83.06 ± 8.79	*F* = 0.078	0.925
	Senior nurse	339	9.81 ± 1.65^a^			43.11 ± 4.55^a^			82.72 ± 9.25		
	Supervisor nurse and above	139	9.72 ± 1.42^a^			43.84 ± 3.98^a^			82.92 ± 8.02		
Number of IHT	0–5 times	455	9.81 ± 1.61	*t* = 2.54	0.011	42.38 ± 4.57	*t* = −5.00	<0.001	82.39 ± 9.22	*t* = 2.54	0.011
	6 and above	157	9.43 ± 1.60			44.47 ± 4.30			84.13 ± 7.67		
Received ITH education or training	No	63	9.84 ± 1.46	*t* = 0.64	0.524	40.52 ± 3.64	*t* = −4.29	<0.001	82.00 ± 8.61	*t* = −0.79	0.428
	Yes	549	9.71 ± 1.63			43.20 ± 4.52			82.94 ± 8.91		
Theoretical teaching in departments	No	74	9.85 ± 1.32	*t* = 0.83	0.405	42.73 ± 4.24	*t* = −0.93	0.354	81.78 ± 8.86	*t* = −1.20	0.232
	Yes	475	9.68 ± 1.67			43.25 ± 4.55			83.12 ± 8.91		
Emergency drills in clinical departments	No	146	9.26 ± 1.62	*t* = −3.85	<0.001	42.41 ± 4.83	*t* = −2.40	0.017	82.66 ± 9.50	*t* = −0.34	0.667
	Yes	403	9.86 ± 1.60			43.46 ± 4.37			83.03 ± 8.69		
Hospital‐level training	No	253	9.44 ± 1.72	*t* = −3.54	<0.001	42.33 ± 4.46	*t* = −4.17	<0.001	82.30 ± 9.57	*t* = −1.54	0.124
	Yes	296	9.93 ± 1.50			43.91 ± 4.42			83.48 ± 8.28		

*Note*: Different upper letters (a,b and c) in the same column indicate statistical significance among groups (p<0.05; Anova and Tukey post test). And the same letters means no sianificance (p>0.05).

### 3.5 Factors affecting nurses' KAP scores on IHT of critically ill patients

KAP scores were taken as dependent variables to explore the influencing factors of nurses' KAP on IHT of critically ill patients. Additionally, the statistically significant factors in univariate and correlation analyses were taken as independent variables and multiple linear stepwise regression analyses were carried out. The assignment methods of independent variables are shown in Table 4 and the scores of KAP were entered with the original values.

**TABLE 4:** Independent variable assignment table.

Independent variable	
Departments	ICU and Emergency room = 0, Other departments = 1
Gender	Men = 0, Female = 1
Age	20–29 = 0, 30 and above =1
Years of nursing practice	1–2 years = 0, 3–5 years = 1, More than 6 year = 2
Job grade	N0 = 0, N1 = 1, N2 = 2, N3 and above = 3
Professional title	Staff nurse = 0, Senior nurse = 1, supervisor nurse and above =2
Number of IHT	0–5 times =0, 5 times and above = 13
Received ITH education or training	No = 0, Yes =1
Emergency drills in clinical departments	No = 0, Yes =1
Hospital‐level training	No = 0, Yes =1

Variables that were associated with the scores of transfer knowledge were the scores of transfer practice (*β* = 0.179, *p* < 0.001), different departments (*β* = 0.098, *p* < 0.05) and the scores of transfer attitude(*β* = 0.101, *p* < 0.05) (*F* = 6.68, *p* < 0.001), accounting for 6.9% of the total variation (adjusted *R* ^2^ = 0.069). The score of practice (*β* = 0.395, *p* < 0.001), number of IHT (*β* = 0.124, *p* < 0.05) and received hospital‐level training (*β* = 0.216, *p* < 0.05) had statistical significance on the nurses' attitude scores (*F* = 20.47, *p* < 0.001), accounting for 26.0% of the total variation (adjusted *R* ^2^ = 0.260). Furthermore, the score of the attitude (*β* = 0.401, *p* < 0.001) and transport knowledge (*β* = 0.147, *p* < 0.001) had statistical significance on the nurses' practice (*F* = 39.68, *p* < 0.001), accounting for 20.2% of the total variation (adjusted *R* ^2^ = 0.202). Details of the multiple linear regression model are presented in Table 5.

**TABLE 5:** Multiple linear regression examining factors affecting nurses' knowledge, attitude and practice of IHTof critically ill patients.

Dependent variable	Independent variable	Regression weight	SE	Standardised regression weights	*t*‐value	*p‐value*	*R* ^2^	Adjusted R2
The score of knowledge	Constants	4.715	0.881		5.353	0.000	0.081	0.069
	The score of Practice	0.032	0.008	0.179	4.114	0.000		
	Department	0.354	0.165	0.098	2.147	0.032		
	The score of Attitude	0.035	0.016	0.101	2.228	0.026		
The score of Attitude	Constants	22.931	2.729		8.402	0.000	0.273	0.260
	The score of practice	0.204	0.019	0.395	11.000	0.000		
	Number of IHT	1.298	0.438	0.124	2.967	0.003		
	Hospital‐level training	0.843	0.352	0.216	2.392	0.017		
The score of Practice	Constants	44.468	3.851		11.547	0.000	0.207	0.202
	The score of attitude	0.776	0.073	0.401	10.693	0.000		
	The score of knowledge	0.814	0.205	0.147	3.973	0.000		

## 4 DISCUSSION

The results of this survey show that only 32.8% of the clinical nurses were reported to have a high level of knowledge, indicating that the nurses' knowledge of IHT for critically ill patients still has room for improvement, consistent with the findings of other studies (Hu et al., 2021). Among the items in the knowledge questionnaire of this study, the top three items were ‘Preparation of stuff before IHT’, ‘Evaluation content before IHT’ and ‘Administration of medication during transportation’ with scores of (0.96 ± 0.20), (0.94 ± 0.24) and (0.94 ± 0.25), respectively. This may be related to applying and optimiseng the transfer checklist for critically ill patients in the hospital. In previous studies (Williams et al., 2020), a checklist was useful in improving safety in transporting a critically ill patient population. The above three items are required in the transfer checklist, used as a check tool for nurses before transfer. Moreover, clinical nurses evaluate and complete the preparation work for critically ill patients before transfer according to the items in the checklist one by one.

The three Items with the lowest scores were ‘You are transporting a mechanically ventilated patient to CT Scan and you do not have a portable mechanical ventilator. How do you ensure continuous ventilation for the patient?’, ‘What is the acceptable minimum level of oxygen in the oxygen cylinder for safe transport of the critically ill patient?’ and ‘When you are ready to transport critically ill patients, which of the following personnel will you inform?’, with scores of (0.45 ± 0.50), (0.32 ± 0.47) and (0.25 ± 0.43), respectively. A possible reason was that the nurses included are not just working at the ICU. Thus, these results suggest that nurses didn't have enough comprehensive knowledge about the IHT of critically ill patients and could not skillfully apply it to clinical transport practice. The scores of the IHT knowledge in the ICU and Emergency room (9.40 ± 1.62) were lower than in Other departments (9.84 ± 1.59). In general, nurses in the ED and ICU should be more proficient in the knowledge of transporting critically ill patients. A possible reason was that some of the nurses in the ED and ICU were so busy that their knowledge was insufficient. However, nurses in the ED and ICU are necessary personnel in charge of IHT for critically ill patients. They must have sufficient IHT knowledge, clinical experience and emergency response ability.

These results showed that 89.7% of nurses had been trained in the knowledge of critical patient transport, 77.6% of nurses had completed theoretical teaching in their departments and 48.4% had participated in hospital‐level training. However, this was not apparent from the survey results. This shows that the current training content and form can not meet the needs of nurses' transport training for critically ill patients. Relevant guidelines (Critical Care Medicine Branch of Chinese Medical Association, 2010) also point out that the correct training of transport persons is essential to ensuring the safe transport of critically ill patients. However, the standards recognised by various hospitals are different in China, potentially resulting in reduced training and quality of nurses. The patient's condition is also a dynamic process during IHT, requiring nurses to have professional emergency handling abilities and comprehensive evaluation abilities. The findings of existing studies suggest that experiential learning enables trainees to acquire knowledge and master technologies used in practice to enhance their skills in teamwork, critical thinking and communication (Xie et al., 2020). Therefore, we should gradually apply experiential training to all fields of nursing education. At the same time, it also reminds managers to develop a multi‐level experiential training program that is systematic, comprehensive and demand‐oriented. Additionally, the training in emergency ability should be emphasised to improve clinical nurses' ability to evaluate and monitor patients' condition, deal with emergencies and reduce the incidence of AEs of IHT in critically ill patients.

The study observed that the positive rate of nurses' transport attitude towards critically ill patients was 36.9% and 61.1% had neutral attitudes, indicating that nurse's attitude towards IHT of critically ill patients still needs to be improved. Further analysis of each item showed that ‘I have no confidence in transporting critically ill patients because I haven't received enough training and experience related to IHT’, ‘One never finds time to prepare for transport of patients because everything is done in a rush’ and ‘I fear transporting critically ill patients because we have had many adverse events during patient transportation’ had low scores. Notably, these results echo Hu et al. (2021), in which the shortage of human resources is proposed to lead to AEs when critically ill patients are transported. Another cross‐sectional study showed that the AEs encountered by nurses during IHT of critically ill patients would also reduce their confidence (Jia et al., 2016) and increase their pressure (Ringdal, Chaboyer & Stomberg, 2015; Song et al., 2022). In the future, we can explore the intervention measures to reduce nurses' psychological burden in critically ill patients' IHT. The study observed that the number of IHT and received hospital‐level training statistically impacted the attitude scores. The results showed that the number of IHT>5 and receive hospital‐level training in the hospital, the more positive the nurses' attitude towards the IHT of critically ill patients would be. In the study, ICU (58.2%) was the department with the largest number of people participating in hospital‐level training and emergency nurses (63.2%) and ICU nurses (60.0%) had the highest frequency of number of IHT>5 in 1 year. This shows that there were more opportunities to participate in the practice of transporting critically ill patients and related training in the ED and ICU. Therefore, hospital administrators should provide adequate training for nurses, especially in other departments except ED and ICU and make emergency plans and drills in advance for accidents in the transfer process (Bergman et al., 2020). In doing so, they can enhance nurses' confidence in the IHT of critically ill patients, promote clinical nurses to establish a correct and positive attitude and ensure the transfer safety of critically ill patients.

Compared with the attitude score, the score of practice was higher. Despite the lack of IHT knowledge and transport experience, most nurses had a positive or neutral attitude towards it, which showed that nurses could realise the importance of transporting critically ill patients and seriously implement its related processes. The results indicate that the better the nurses master the relevant knowledge, the more positive their attitude and the more standardised their transport behaviour. Thus, this suggests that nurses's knowledge level of IHT can affect their attitude and behaviour. It should be noted that because the score of IHT practice behaviour in this study is not in the form of on‐site observation, it may lead to a high score if nurses score it themselves. The study results showed that the last two scores of the practice dimension items were ‘I made a comprehensive evaluation of the transshipment work after IHT’ and ‘I know the area where I can get first‐aid equipment on the IHT route’. It means that nurses have a low awareness of the first‐aid equipment available during IHT, which suggests that managers should pay attention to training first‐aid equipment acquisition methods and managing eye‐catching signs. There is no comprehensive evaluation summary after IHT, which suggests that managers should give weight to the quality control of the whole IHT process. The summary and evaluation after IHT can provide a basis for the subsequent improvement of the IHT procedure and evaluate the rationality of members' composition, the effectiveness of communication and the pertinence and predictability of planned measures. In addition, effective assessment and grading of the risk of patient transport and prompt preparation according to the actual situation can aid the reasonable allocation of medical resources and further improve the safety of transport, thereby enabling better treatment of critically ill patients (Ling et al., 2023).

### 4.1 Limitations

We developed and tested a questionnaire strictly focused on nurses' KAP towards IHT. Furthermore, our results might not be generalisable because this was a cross‐sectional study with convenience sampling. This survey is only carried out among clinical nurses in one hospital and the sample size is limited, which needs further expansion. Finally, desirability bias might have existed in our study because we used a self‐reporting questionnaire.

## 5 CONCLUSIONS

The findings indicate a clear need for clinical nurses' knowledge of IHT of critically ill patients, especially in the ED and ICU. Furthermore, nurses need to be more active in the transfer of critically ill patients. The findings remind managers to gradually apply new training methods to nursing education. At the same time, it also reminds managers to develop a multi‐level training program that is systematic, comprehensive and demand‐oriented.

## AUTHOR CONTRIBUTIONS

Study concept and design: HZ and JD; Acquisition of data: ZX.; Analysis and interpretation of data: YY.; Drafting of the manuscript: HZ., JD. and YY. All authors contributed to revising the manuscript critically for important intellectual content and final approval of the version to be submitted. JD and YY contributed equally to the work.

## FUNDING INFORMATION

This study was internally funded by the Medical Scientific Research Foundation of Zhejiang Province, China (2022KY873).

## CONFLICT OF INTEREST STATEMENT

The authors declare that they have no known competing financial interests or personal relationships that could have appeared to influence the work reported in this article.

## Data Availability

The data that support the findings of this study are available from the corresponding author upon reasonable request.
